# Supplementary material for: Factors associated with miscarriage in Nepal: Evidence from Nepal Demographic and Health Surveys, 2001–2016
Source: PLoS One. 2024 Apr 29;19(4):e0302001. doi: 10.1371/journal.pone.0302001 (PMC11057729; doi:10.1371/journal.pone.0302001)
Supplement: Attachment — Submitted filename: JS REVIEW.docx [file pone.0302001.s001.docx]

Thank you for providing me with the opportunity to review the manuscript titled "Factors Affecting Miscarriage in Nepal: Evidence from Nepal Demographic and Health Surveys, 2001-2016." (PONE-D-23-17092). After carefully reading the manuscript, I would like to recommend the following changes for the improvement of the article.

1. The authors are advised to specify "The study found that the couple's age —" (Line 84). Proper citation is needed here.
2. The authors are asked not to repeat the same things. The smoking behaviour and miscarriage are represented in lines 90 and 97.
3. The calculated percentages in Table 1 are not clearly mentioned whether these are weighted or unweighted. Authors are advised to mention it.
4. Authors are asked to provide the rationale behind considering few variables like ecological zone, wealth index, sex of household head etc. in this study.
5. The discussion section should focus on the explanation of the major findings and their implications. So, the authors are advised to refrain from presenting the numerical results.
