# Supplementary material for: Factors associated with miscarriage in Nepal: Evidence from Nepal Demographic and Health Surveys, 2001–2016
Source: PLoS One. 2024 Apr 29;19(4):e0302001. doi: 10.1371/journal.pone.0302001 (PMC11057729; doi:10.1371/journal.pone.0302001)
Supplement: Attachment — Submitted filename: Response to Reviewers.docx [file pone.0302001.s003.docx]

**Rebuttal Letter**

The authors would like to thank the reviewers for their fair reviews and detailed list of constructive Suggestions. We greatly appreciate the opportunity to submit a major revision of our paper for your renewed consideration. We have given more detail based on comments we are receiving from you. Below, we respond to reviewers’ comments and point to the corresponding changes we made in the paper.

**Reviewer (R3) concerns:**

Reviewer #3: This is a great piece of work in a topic where limited research are done, especially in resource poor settings like Nepal. This manuscript may benefit from some minor revisions as described below.
Abstract
Methods: write factors associated instead of significant factors
Results: please remove the first sentence to reflect more on your study title.
Conclusion: please be specific with your results when you draw the conclusion

Introduction: Please look at English grammar throughout.
Methods: Please combine exposure variables and confounding variables into one heading under study factors.
Analysis: remove ‘Method of analysis’ and replace with ‘Statistical analysis’
Figure/table headings: Please re-write your fig/table headings (for example: Table 2: Factors associated with miscarriage in Nepal, NDHS 2001-2016)
Please be consistent with the choice of word. For example, if your title is factors associated with miscarriage, please use them throughout rather than replacing it with word such as predictor.

**Our responses**

**Abstract**

**Methods**: We have changed factor associated instead of significant factors in methods section as per your suggestion.

**Results**: We removed first sentence from result section.

**Conclusion**: We revised our prior conclusion and were more specific with the findings of our study in new revised manuscript.

**Introduction**: We worked with a professional paper editor to revise our article and make grammatical and sentence structure corrections.

**Methods**: We made the changes you suggested put in one heading under study factors.

**Analysis:** We made correction and replacing methods of analysis in analysis section.

**Figure/table headings**: In response to your comments, we reworked our figure/table title to be shorter and more concise.

**Please be consistent with the choice of word. For example, if your title is factors associated with miscarriage, please use them throughout rather than replacing it with word such as predictor.**

We have tried to become consistent in our word choice throughout the article. We changed the word predictor to the factor associated with it to maintain consistency across the study.

**Reviewer #4 :( concerns)**

Reviewer #4: The paper have been designed with the help of DHS 2001-2016, but the recent demographic data of Nepal (DHS-2022) is available and the analysis can be done with the help of recent published data which will have more applicability in terms of present date. Therefore, the latest data should be incorporated in the study. More factor variables (obesity status of women) can be used as explanatory variables against miscarriage. The study shows that miscarriage rate of Nepal from 2001 to 2016 is increased by 4.2 percentage points. Proper explanation is very necessary regarding the gradual increasing of miscarriage rate from 2001 to 2016, in spite of having improved medical facilities day by day.

**Our Responses:**

The authors would like to thank you for your suggestions for the study. Of course, the most recent data DHS-2022 report is already accessible. The study used data from the NDHS 2001 to the NDHS 2016 since the study is based on the Millennium Development Goal era, which is from 2000 to 2015, the finding would assist to establish strategies for achieving Sustainable Development Goal (SDG) Goal-3 to ensure healthy lives and promote well-being for all at all ages. And also we have sufficient sample size around 26,376 which is sufficient enough to calculate power. It is like similar proportion of Miscarriage in DHS 2022 in Nepal is about 9.4% almost near to equal level of proportion with DHS 2016**.** The data was released after the COVID-19 pandemic, therefore it is possible that it was impacted by the COVID-19 pandemic. Thank you for your suggestion; I will include it in a future publication as I am preparing my next paper on miscarriage based on DHS 2022 data.  The variable obesity status of women has previously been proven as a risk factor for miscarriage in Nepal by Pramesh et al on an association between obesity and miscarriage in Nepal based on data from 2001 to 2016, we were unable to include it in our study. The DHS data shows that miscarriage is increasing in Nepal and there are various reasons associated with it. Limited access to maternal health care services, Poor quality of care, malnutrition, poor maternal health are the factors contributing to increased miscarriage in Nepal, however this is still an area that needs to be explored.
