# Supplementary material for: Factors associated with miscarriage in Nepal: Evidence from Nepal Demographic and Health Surveys, 2001–2016
Source: PLoS One. 2024 Apr 29;19(4):e0302001. doi: 10.1371/journal.pone.0302001 (PMC11057729; doi:10.1371/journal.pone.0302001)
Supplement: Attachment — Submitted filename: Response to Editor.docx [file pone.0302001.s004.docx]

**Rebuttal Letter**

The authors would like to thank the editor for their fair reviews and detailed list of constructive suggestions. We greatly appreciate the opportunity to submit a minor revision of our paper for your renewed consideration. Below, we respond to the editor's comments and point to the corresponding changes we made in the paper.

**Journal Requirements:**

Please review your reference list to ensure that it is complete and correct. If you have cited papers that have been retracted, please include the rationale for doing so in the manuscript text, or remove these references and replace them with relevant current references. Any changes to the reference list should be mentioned in the rebuttal letter that accompanies your revised manuscript. If you need to cite a retracted article, indicate the article’s retracted status in the References list and also include a citation and full reference for the retraction notice.

Our Response:

We have reviewed the list of references. There are no any references that have been retracted. One of the references that we cited earlier have been removed.
